# The experiences of autistic adults who were previously diagnosed with borderline or emotionally unstable personality disorder: A phenomenological study

**DOI:** 10.1177/13623613241276073

**Published:** 2024-09-11

**Authors:** Bruce Tamilson, Jessica A Eccles, Sebastian C K Shaw

**Affiliations:** 1 Brighton and Sussex Medical School, UK

**Keywords:** Autistic, borderline personality disorder, emotionally unstable personality disorder, interpretive phenomenology, lived experience, qualitative

## Abstract

An increasing number of studies are investigating the links between autism and borderline personality disorder. Studies report overlapping differences and the challenges in differentiating between these two diagnostic labels. In practice, there are many people, especially autistic women, who feel that they were misdiagnosed with borderline personality disorder. This study aimed to explore the experiences of autistic adults who were previously diagnosed with borderline personality disorder. This is an interpretive phenomenological study. Data were collected using one-to-one, semi-structured interviews. Interview audio-recordings were transcribed and analysed using an interpretive phenomenological analysis. Ten people participated. All recalled autistic differences since childhood that went unnoticed. In most cases, borderline personality disorder was felt to have been a misdiagnosis. This misdiagnosis carried stigma, introduced diagnostic overshadowing and led to harmful experiences for our participants. While they did not identify with the diagnosis, they felt powerless to challenge it. In contrast, receiving an autism diagnosis was ‘life changing’. While this did not solve everything for them, it was deeply validating. It also allowed them to be their true authentic selves, shifting the focus away from ‘treatment’ and changing who they are to suit others, towards acceptance of their differences and neurotype. This significantly improved their mental health.

**Lay Abstract:**

Autistic people face many barriers to receiving an autism diagnosis. Often, they may be misdiagnosed with borderline personality disorder instead. For our study, we interviewed 10 autistic adults who had previously been diagnosed with borderline personality disorder. This helped us to better understand their experiences. They explained how borderline personality disorder is quite stigmatised and may suggest that people are to blame for their differences in behaviour. They found the treatments they had to try for borderline personality disorder to be harmful. For example, these treatments promoted ‘masking’. Previous research showed that masking can be harmful for autistic people, linking it to risk of suicide. This diagnosis also led to healthcare professionals neglecting them and discounting their beliefs. Once they were diagnosed with borderline personality disorder, it was hard to access an autism assessment. When they did receive their autism diagnoses, this was much more positive. This diagnosis was validating. It also improved their mental health, as they were no longer expected to mask – their differences were now accepted. They still felt that autism was stigmatised in society. However, this was very different to the stigma around borderline personality disorder. They felt autism stigma was more about their competence as people, whereas borderline personality disorder stigma was about how they were broken and might be harmful to others. This study is important because it allows their stories to be heard by researchers and healthcare professionals alike. Adding their voices helps to humanise them, promoting positive change in mental health services. More research is now needed.

## Introduction

Borderline personality disorder (BPD) is also known as emotionally unstable personality disorder (EUPD) in the United Kingdom – as per International Classification of Diseases, tenth revision (ICD-10), BPD is a type of EUPD. Both autism and BPD/EUPD can manifest as similar differences in the way people experience and interact with the world (May et al., 2021). Commonalities may include differences around executive functioning, social preferences, intimacy, emotions and socio-occupational status (Dudas et al., 2017; May et al., 2021; Strunz et al., 2015). Studies have also shown that autistic people and those diagnosed with BPD/EUPD are also less likely to be perceived as agreeable by others (Strunz et al., 2015). This is further supported by the fact that experiencing challenges in relationships with others is commonly presented as a distinguishing factor for both diagnostic labels (Carey et al., 2019). Furthermore, self-injurious behaviours and suicidal ideation/attempts are often experienced by both autistic people and those diagnosed with BPD (American Psychiatric Association, DSM-5 Task Force, 2013; Hedley et al., 2018; Ingenhoven, 2020). As such, clinician bias can result in frequent misdiagnoses and missed diagnoses when overlapping diagnostic criteria are present (May et al., 2021). The impact of clinicians’ knowledge, experience, beliefs and biases on psychiatric diagnoses is well-acknowledged in literature. This issue, particularly concerning BPD and autism, is highlighted in the Delphi study by Cumin et al. (2022). In addition, the Royal College of Psychiatrists and National Health Service (NHS) England have recognised the lack of autism knowledge among clinicians and have initiated national training programmes to address this gap.

Studies show that 70% of autistic people have been diagnosed with co-occurring conditions, including personality disorders (Mosner et al., 2019). When people are assessed by mental health services in adulthood, they often receive a diagnosis of personality disorder (Anckarsäter et al., 2006). Furthermore, when adult women get diagnosed as autistic, the most common de-diagnosis is a personality disorder (Kentrou et al., 2021). Autistic women diagnosed with BPD also often have their autistic status missed (Hull et al., 2017). Intersectional considerations like this are important to understand diagnostic reasoning processes here. Non-stereotypical presentations of autism are widely discussed in the published literature (Hull et al., 2020). However, this predominantly focuses on cis women. There is a need for research into the autism presentation in the LGBTQ+ (lesbian, gay, bisexual, transgender, questioning (or queer) and more) population, ethnic minorities and people with intellectual disability (ID), for example.

Van Wijngaarden et al. reported that the male-to-female ratio of autism is 2:1 in people with ID and 9:1 in those without ID (Van Wijngaarden-Cremers et al., 2014). The difference in gender ratio was thought to be due to the female protective effect theory, where females require higher level of genetic and environmental ‘risk’ than males to show the same level of autistic features (Robinson et al., 2013). This has since been refuted through social explorations around diagnostic reasoning. For example, it has been argued that this discrepancy may be due to the underdiagnosis of autism in females (Wing, 1981), alongside overdiagnosis with BPD/EUPD (Skodol, 2003). All this makes accurate diagnosis, especially in females, challenging for many clinicians. In turn, this may hold significant psychological and social implications for people receiving these diagnostic labels. Receiving a correct diagnosis can bring a sense of relief for autistic people, which empowers them to build an authentic sense of self, while validating the challenges they have experienced (Belcher et al., 2023; Bulluss & Sesterka, 2020; Hickey et al., 2018). Furthermore, missing an autism diagnosis can be harmful. This is associated with high rates of stress, exhaustion, anxiety, depression and death by suicide (Fusar-Poli et al., 2022). It is, therefore, vital that we understand the experiences of people who have been through this journey.

This study aims to explore the experiences of autistic adults who have previously received a BPD/EUPD diagnostic label. This aims to help improve our understanding of this experience and the potential consequences of misdiagnosis, to provide insight to clinicians about individual experiences, to improve the lives of those people experiencing misdiagnosis with BPD/EUPD.

## Methods

### Paradigmatic lens

We conducted this study from a social constructionist perspective. Social constructionism suggests that our realities are created through social interactions with others. It combines a relativist view of existence (ontology) with a subjective view of knowledge (epistemology), promoting interpretive methods in research (Harris, 2006). This philosophical standpoint posits that ‘*meaning is not inherent and that meaning is central to social life*’ (Harris, 2006). As such, this lays the foundations for critical and interpretive research approaches.

### Methodology

An interpretive phenomenological approach was adopted. This is a qualitative methodology, focused on the study lived experience (Shaw & Anderson, 2018a). Drawing on hermeneutics and ideography, this steps beyond descriptive approaches to embrace creativity, subjectivity and interpretation – from participants and researchers (Shaw & Anderson, 2018a; Smith et al., 2021). As previously explained, interpretive phenomenology

‘acknowledges the fact that we, as humans, are bound in our thinking by our position, in the world, in society, and in history, and it would be impossible to (and pretentious to assert that we could) shed all of these influences on our thinking’. (Smith et al., 2021)

This approach therefore seeks to understand the shared experiences by actively encouraging an interpretive, sense making process – in particular, the researchers’ interpretations of participants’ interpretations of their lived experiences (Smith et al., 2021).

### Community involvement and positionality

Our own positionality influenced our methodological choices. S.C.K.S. has extensive experience in using interpretive phenomenology in similar areas, for example, to explore the experiences of autistic (Shaw et al., 2023), dyslexic (Shaw & Anderson, 2018b; Shaw, Hennessy, & Anderson, 2022), dyspraxic (Walker et al., 2021) and attention deficit hyperactivity disorder (ADHD; Godfrey-Harris & Shaw, 2023) medical students.

Our team includes both autistic and non-autistic authors. For example, the Principal Investigator (S.C.K.S.) is autistic. Our team are also all medical doctors, bringing certain professional experiences and perspectives. Despite our partial insider status, none of us have been diagnosed with BPD/EUPD. Through his own autistic status, SS recognised the importance of this study topic from a social justice perspective for the autistic community. He had frequently heard longing from those with lived experience in this area to find a study like this, to validate their experiences. To that end, an autistic colleague who had previously been diagnosed with BPD was involved in the conception of the project, including supporting the detailing of our methods. Furthermore, a colleague specialising in mental health for people with a diagnosis of BPD also supported the early phases of project design. This holistic focus, from multiple perspectives, was vital to our approach, to ensure the project aims, ethos and methods would be in line with community priorities (Pukki et al., 2022) – helping to tackle epistemic injustice (Shaw et al., 2023).

### Ethical approvals

The Brighton and Sussex Medical School Research Governance and Ethics Committee approved this study (reference: ER/BSMS9BCV/1).

### Sampling, recruitment and consent

This study applied purposive sampling approach. Participants had to be autistic adults (including self-diagnosed) who had a previous diagnosis of BPD/EUPD. Participants had to speak English and had to be based in the United Kingdom.

Announcements were posted on Twitter. Interested people contacted B.T. over email for more information. A participant information sheet was then provided. B.T. received audio-recorded informed consent from each participant prior to data collection.

### Data collection

One-to-one interviews were used to collect data. We used a semi-structured approach, facilitating both focus and flexibility for individual participant needs. An interview topic guide (see Supplemental material) was constructed iteratively between B.T. and S.C.K.S., grounded in their own experience, knowledge from clinical practice and a literature review. This was further revised following feedback from trusted colleagues, including one with direct lived experience.

Participants were video interviewed via Microsoft Teams. These were audio-recorded. Recordings were transcribed verbatim by B.T. and cross-checked by S.C.K.S. for accuracy.

### Data analysis

This study followed interpretive phenomenological analysis (IPA), adapted from Smith et al. (2021). This took place one transcript at a time. B.T. first immersed himself. He then read through the transcript again to make exploratory comments. Then, these comments were constructed into experiential statements. Then, these statements were converted into personal experiential themes (PETs). One each transcript was analysed individually, all PETs were reviewed to construct group experiential themes (GETs). Throughout this process, B.T. regularly checked in with S.C.K.S. to discuss the analysis. This facilitated a hermeneutic cycle (Smith et al., 2021) and resulted in an increasingly interpretive analysis.

Embracing creativity can be a useful tool within interpretive phenomenology, promoting non-cognitive (e.g. emotional) knowledge advancement (Flood, 2010). To this end, we also used I-Poems to supplement our analysis (Gilligan & Eddy, 2017). An I-Poem in qualitative research compiles sentences starting with ‘I’ from a participant’s narrative into a poem to highlight their personal experiences and perspectives. However, taking an adapted cross-sectional approach to these, B.T. used this analytic method to group and portray emotional aspects of experiences across participants. Here, we shall include an I-Poem to portray the emotional impact of receiving a BPD/EUPD diagnosis.

## Results

### Participants

Ten people were interviewed (see Table 1). The mean age of the participants was 34. The mean age for receiving a diagnosis was 22 for BPD/EUPD and 29 for autism. All participants were from England.

**Table 1.** Participant demographics.

	Duration of the interview in minutes	Age	Gender identity	Region of England	Age at diagnosis of BPD	Age at diagnosis of autism (NHS or private)
P1	63	30	Female	South East	28 (Private)	30 (NHS)
P2	71	26	Female	East	19 (NHS)	20 (Private)
P3	51	27	Female	North West	19 (NHS)	20 (NHS)
P4	55	43	Female	South East	27 (NHS)	19 and 40 (NHS)
P5	57	31	Questioning	London	17 (NHS)	15 and 21 (NHS)
P6	66	52	Female	North	20 (NHS)	52 (Private)
P7	57	24	Female	South East	14 (NHS)	18 (NHS)
P8	102	36	Female	South East	30 (NHS)	34 (NHS)
P9	54	24	Female	East	20 (Private)	20 (NHS)
P10	54	44	Female	South East	20 (NHS)	39 (NHS)

BPD: borderline personality disorder; NHS: National Health Service.

### Analysis overview

**Figure 1.**
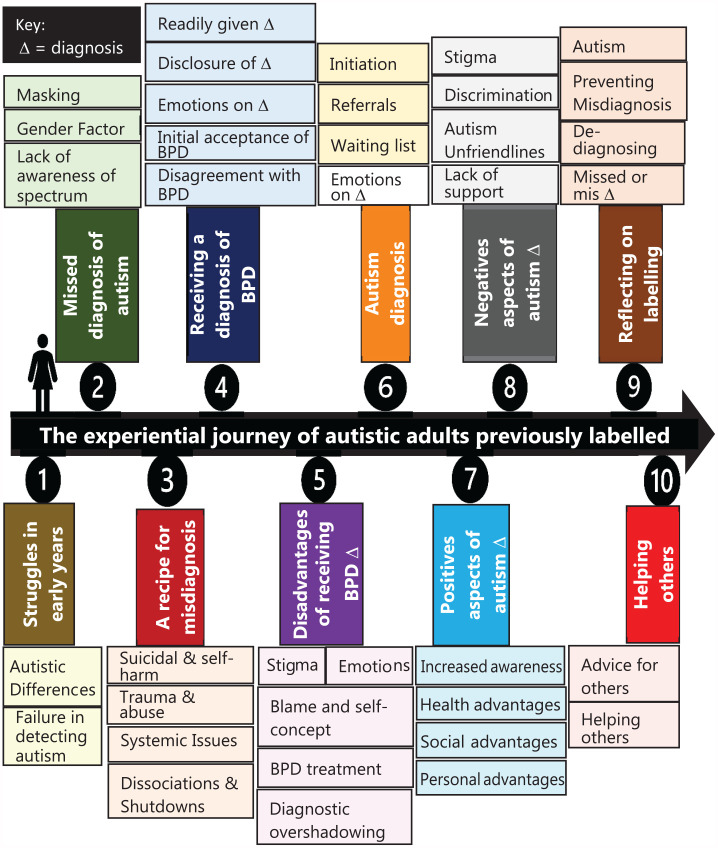
The thematic journey of our participants.

Our analysis resulted in 10 constructed GETs. Through our interpretation, it became clear that their interconnection reflected the personal journeys of our participants, from early life through to present day (see Figure 1). Accordingly, our results are presented in this way.

### GET1: struggles in early years

All participants embodied autistic differences lifelong. The recognition of such differences came to light through self-recognition, or suggestions of close others (e.g. family, peers or schoolteachers).

‘*The school noticed that I was a bit odd*.’. (P5)

Most felt ‘different’ from their peers at a young age, recalling that they did not fit in socially. These differences resulted in experiences of anxiety around non-autistic people, resulting in a scarcity of friends.

‘*Well, I knew I didn’t fit in, and I was alien, but these kids could recognize it and I was always bullied . . . . I did not belong*’. (P2)

Despite self-awareness of their differences, none were diagnosed as autistic in early childhood. This was even the case for participants who had contact with mental health services for co-occurring conditions.

‘*Years later when I saw the notes. . . I was surprised they didn’t pick up that I might be autistic . . .* ’. (P1)

### GET2: missed diagnosis of autism

This trend of missed diagnosis continued into adulthood for most. Most participants discussed how they had learned to ‘mask’ their autistic differences in order to survive in this world.

‘*I was probably like quite a big masker* . . . ’. (P1)

P2, for example, outlined how they ‘*pretend to fit in*’ – explaining that this is ‘*not out of choice*’, but rather a ‘*survival technique*’ by mimicking social cues and body language. While this helped participants to navigate social exclusion and stigma, this came at a cost.

‘*It’s very exhausting and draining*’. (P2)

When making sense of their missed diagnosis, participants discussed the impact of gender. They attributed perceived ‘female’ gender as a key factor in why their autistic identities were overlooked and/or missed by others.

‘ . . . *if I was a boy . . . it probably would have been picked up on while I was still in primary school*’. (P7)

Furthermore, most participants’ experiences had led them to feel that the mental health system itself was biased against non-cis-male gender identities, among a variety of other intersectional factors.

‘*[There] is so much bias when it comes to being assessed for autism . . . the whole test is racist, classist, sexist, and . . . everything*’. (P2)

Experiences with mental health services had left participants feeling despondent. Making sense of these experiences, participants passionately reported a lack of autism awareness among professionals – leading them to overlook its spectrum nature, in favour of traditional stereotypes.

‘*They have no idea about autism, like none*’. (P5)

Lack of childhood diagnosis was, in itself, experienced as a barrier to adult diagnosis. Participants reported how clinicians used this as evidence against considering an autism diagnosis, discounting participants’ beliefs and experiences.

‘*Well you’ve got this far through life without having a diagnosis of autism . . .* ’. (P2)

‘*She laughed and said you’re not autistic*’. (P10)

### GET3: a recipe for misdiagnosis

Suicidality or self-harm experiences mostly resulted in inpatient admissions, as well as being the entry point into services. Most reported the reason for self-harming as hopelessness or a way of coping. However, making sense of their experiences, participants felt that their initial presentations centring around suicide or self-harm biased clinicians’ diagnostic reasoning.

‘*Doctors . . . often think ‘oh, it’s for attention’*’. (P2)

Participants explained how this bias led directly to their misdiagnosis with BPD/EUPD.

‘*I think my diagnosis of BPD was purely based on the fact that I was self-harming*’. (P4)

Further considering the meaning behind their misdiagnoses, participants reflected on their experiences of autistic shutdowns, exploring how these were labelled as dissociative experiences when seen through a BPD lens. Participants used various terms to describe the experience: ‘*dissociation*’, ‘*brain just goes offline*’, ‘*just like a fuse goes*’, ‘*shutdown*’, *or* ‘*zone-out*’.

‘*I used to have periods where I just really zone out which from a personality disorder viewpoint was like the tick box for dissociation*’. (P9)

Experiences of trauma were also key to participants’ sense making processes. Most related current mental health challenges to traumatic childhood experiences.

‘ . . . *just an undiagnosed autistic person who had trauma . . .* ’. (P2)

Participants deeply felt that the current model of care does not address these traumatic experiences.

‘*What I needed was trauma therapy to deal with being assaulted*’. (P4)

Instead, the BPD/EUPD diagnostic label consumed them, creating a two-dimensional entity. Participants felt their individual needs were able to be ignored.

‘*This diagnosis (BPD) is damaging, and it covers up the real issue that we have*. (P2)

‘*It’s like it dismisses all trauma*’. (P4)

### GET4: receiving a diagnosis of BPD

Participants felt that BPD diagnoses were given ‘*too readily*’ (P1) and ‘*easily*’ (P2) by psychiatrists.

‘*I think it’s a lazy diagnosis*’. (P5)

They recalled feeling that their BPD diagnosis was to benefit their psychiatrists and the wider mental health system, rather than themselves.

‘*I would like if you included the benefit for psychiatrists in diagnosing personality disorder*’. (P3)

‘*It’s more cost-effective for them to say your personality disordered than autistic*’. (P5)

Receipt of this diagnosis led to experiences of feeling neglected. Making sense of this, they reported that a BPD diagnostic label overshadowed their individuality, allowing them to receive less appropriate treatment.

‘[It’s] *a get-out clause for not wanting to admit someone*’. (P2)

‘*It’s like . . . we can just basically neglect you . . . and it’s fine because you are personality disordered*’. (P5)

These negative feelings were underpinned by negative experiences of not receiving their diagnoses directly. Participants recalled a reluctance for psychiatrists to disclose the diagnosis to them.

‘*Nobody actually told me*, *nobody*!’. (P6)

Instead, participants found out via clinic letters, discharge summaries, or in passing comments from ward nursing staff.

‘*It was flippantly suggested* . . . ’. (P8)

Initially, participants felt they had no choice but to accept this diagnosis. They explained how they complied to shield themselves, following negative experiences within the services. This decision was also influenced by the perceived position of power of the psychiatrist.

‘*[The] psychiatrist who wrote on my records I had BPD . . . held so much power*’. (P6)

‘*it was more a case of like you will agree to this, or you’ll never get discharged . . . I agreed . . . surrendered*’. (P3)

‘*I was so beaten down at that point, and I had to agree to it*’. (P8)

Despite feigning acceptance, our participants did not truly identify with this diagnostic label. Instead, they did what they felt was needed to survive.

‘*It’s just never fitted*’. (P6)

### GET5: disadvantages of receiving a BPD diagnosis

Participants recalled an aversion to the term BPD itself, let alone its description. On receiving the diagnosis or learning about BPD, participants experienced a wide range of emotions (see Table 2).

**Table 2.** An I-poem exploring the emotions of participants upon receiving a BPD/EUPD diagnosis.

‘*being told that I was personality disordered*’ (P4)	
*I don’t know how to explain that* (P5). *I was so surprised . . .* (P5). *I wanted to throw myself in front of a truck* (P1). ***→ Shock*** I was so ashamed (P8). I shut myself away (P6). I just did not lift my face (P6). I never told anyone (P8). ***→ Shame*** I started getting frustrated (P10). I was horrified (P6). I was annoyed (P5). I felt angry (P6). ***→ Anger*** I did not relate to it (P10). I never felt it related (P6). I didn’t agree (P4). I was so adamant that I didn’t have . . . (P9). ***→ Identity*** I think . . . It’s quite toxic (P6). I just think that’s really insulting (P4). I thought it was offensive (P1). I just felt like a knife to the chest (P3). **→ *Offence***	I felt like. . .it was my fault (P4). I . . . felt like I had a criminal record (P4). I’ve been handed a life sentence (P1). ***→ Guilt*** I was not good enough (P1). I hadn’t tried hard enough (P1). I felt like very core of me was wrong (P4). I thought I am worse (P4). I was led to believe that I’m insane (P6). ***→ Blame*** I could not sleep (P8). I remember crying, just crying, absolutely hysterical crying (P2). I felt sad (P6). I felt . . . awful (P1). ***→ Sorrow*** I felt like I’d lost hope (P4). I was just unable to be helped (P9). I was just so done with life (P2). I don’t know how to say or anything else or other than that I’m sorry . . . (P5). ***→ Despair***

**Figure 2.**
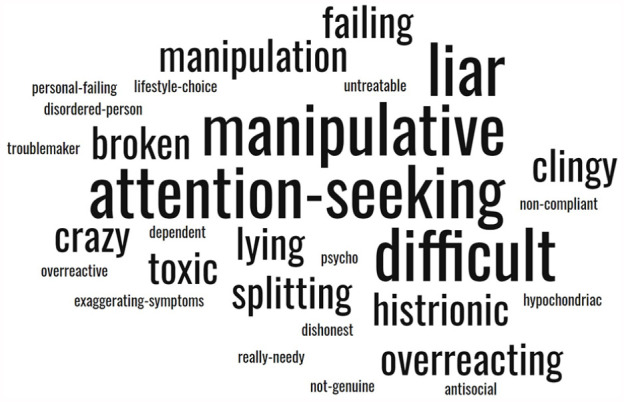
Participants’ perspectives on the stigma others have towards BPD.

Participants felt that the BPD diagnosis was presented in a very stigmatising way. This hurt them deeply, leading them to question how others would subsequently view them (see Figure 2).

‘A *shameful disease*’. (P8)

Reflecting on these potential judgements led to wounds in participants’ self-concept. Who were they now?

‘*I lost my whole sense of self*’. (P3)

‘*It makes you even less certain of yourself and your identity*’. (P7)

Following receipt of this clinical label, participants felt mental health professionals shifted personal blame to them for their actions in times of distress.

‘*Some mental health professionals seem to have the view that people with that diagnosis have more control over their behaviour*’. (P1)

Participants felt ashamed and broken. Subsequently, they did not disclose this diagnostic label.

‘*I don’t imagine a time when I’ll ever be able to tell anyone that I have BPD*’. (P8)

**Figure 3.**
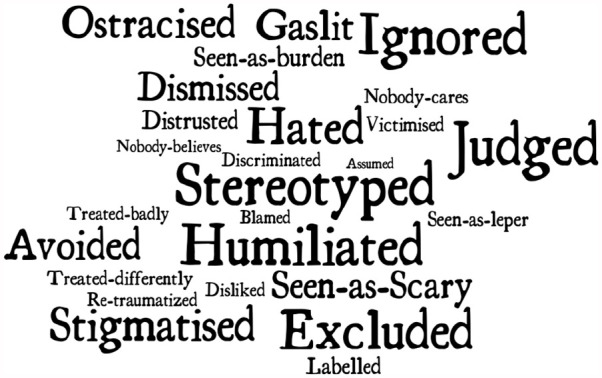
Participants’ perception of treatment of people with BPD.

However, they were unable to avoid disclosure to mental health professionals, who had access to their medical notes. They explained how they felt mental health professionals treated them differently once their BPD/EUPD diagnosis was documented. This felt dehumanising. In most cases, their experiences with mental health services were the most stigmatising of all (see Figure 3).

‘*I think most of the stigma . . ., to be honest, has been from mental health staff like more than anyone else*’. (P4)

‘*Even like doctors . . . they hate people with it*’. (P8)

Subsequently, participants felt disempowered and fearful. They avoided healthcare as a form of self-protection, even when they needed help.

‘*Because of the BPD diagnosis I was too afraid to go through the GP*’. (P6)

All participants experienced inadequate mental health support following diagnosis. Various systemic reasons were attributed.

‘*They said it will be five months to see a psychiatrist*’. (P9)

‘*NHS is so underfunded*’. (P6)

Through their experiences, participants reported that the BPD clinical label allowed services to deny them access. This left them feeling helpless and alone.

‘*Because it’s personality disorder . . . you don’t fit into any service*’. (P5)

In some cases, feelings of helplessness were further reinforced by a sense of futility around their own actions.

‘*If you self-harm . . . ‘she won’t cooperate with the treatment’, . . . if you didn’t self-harm, you are better enough to be discharged*’. (P7)

When they did manage to access help for BPD, subsequent experiences could be overtly harmful.

‘*[It] was more traumatic than any abuse went through my childhood*’. (P3)

In particular, inpatient stays were traumatic due to their unrecognised autistic differences.

‘*You put a body with severe sensory issues into a psychiatric ward*’. (P6)

These experiences led participants to feel that the treatment model around BPD ‘*was not actually fit for purpose*’ (P1) when considering the potential harms for misdiagnosed autistic people. Furthermore, all participants discussed the subsequent diagnostic overshadowing they experienced, delaying formal recognition of their autistic status.

‘*Oh, you’ve got BPD, it’s all down to that . . . any symptoms you have, whether mental or physical or social, all put down to BPD*’. (P7)

### GET6: autism diagnosis

Participants learned about autism from a wide variety of sources, including teachers, family or psychiatrists. Once they realised their autistic identity, however, seeking assessment required them to overcome many barriers; in the forefront was disbelief from professionals.

‘*They didn’t believe me about the autism*’. (P5)

It took significant perseverance to overcome these barriers. In some cases, this required support from others, advocating for their needs.

‘*He ended up referring it because it became abundantly clear that Mum was not going to leave his office until he’d done the referral*’. (P7)

Once referred, the long waits (in some cases, over years) were emotionally turbulent. All the while, living with their BPD diagnostic label instead.

‘*I was waiting and just getting more and more kind of hysterical*’. (P8)

Finally receiving their autism diagnosis was revolutionary for most participants. They experienced waves of relief and validation. In particular, they were able to re-assess their sense of self by this new standard, away from their BPD diagnostic label. One even likened it to the legal system, where BPD had reflected a suggestion of inherent criminality in their very being.

‘*I’d been cleared of like a crime that hadn’t committed . . . it felt like such a relief*’. (P4)

Once the diagnosis had settled in, participants reflected on their experiences. What could have been different for them, had this been identified earlier in their lives? What trauma could they have been spared?

‘*I also felt frustrated and angry that I was misdiagnosed with EUPD*’. (P2)

### GET7: positive aspects of autism diagnosis

Receiving their autism diagnosis had indeed led to the improved life experiences participants had longed for. In part, they attributed this to the improved public understanding and acceptance of autism. This led to more favourable treatment from others, improving their quality of life.

‘*People don’t blame you . . . you’re not at fault*’. (P4)

Having their autistic identity formally validated allowed participants to heal the previous wounds in their self-concept from their BPD/EUPD diagnoses. They were able to accept and be their authentic selves after so many years of masking and complying.

‘*It’s helped me to understand myself a lot more. . . that just explains me*’. (P3)

‘*It gave me almost that justification or validation*’. (P1)

Of particular benefit was a change in ‘treatment’ focus. Unlike with their previous BPD/EUPD diagnosis, participants no longer felt pushed to change themselves to better meet societal expectations. Instead, they were recognised for who they were. This was a liberating experience, which removed a great burden.

‘*BPD is a kind of the pressure to get better from it, whereas the autism is not because it’s just a part of who you are*’. (P7)

‘*I can be my weird self . . . I don’t need to fix anything anymore*’. (P10)

This acceptance led to much improved mental health.

‘*Now I’m discharged from all mental health services as well*’. (P4)

Participants also experienced more acceptance from doctors following their autism diagnosis. They found that their physical health concerns were taken more seriously.

‘*it was only when that (BPD) was removed, and the autism was diagnosed . . . suddenly someone . . . found . . . Crohn’s disease*’. (P7)

Participants also found their autism diagnosis broke down social barriers, where their BPD/EUPD diagnosis had created more. For example, they now received adjustments in their places of study/work.

‘*They’re happy to liaise with university and work if I need reasonable adjustment*’. (P6)

‘*It’s kept me in my job*’. (P3)

In general, reflecting back on their autism diagnosis, this was described as ‘*life-changing*’. (P2)

### GET8: negative aspects of autism

While their autistic diagnostic labels were overwhelmingly more positive than their previous BPD/EUPD ones, there were still issues. Their autism labels still carried stigma. However, this had a different flavour to that surrounding BPD/EUPD. Instead of implications about being a bad or flawed person, participants explained that this stigma centred around questions of their agency or competence.

‘*The reason I don’t overshare the . . . autism diagnosis is worry about people thinking . . .that like I am an idiot or incompetent or not going to be capable of doing things*’. (P7)

Participants still struggled with ableism in large systems, such as universities or healthcare services. Disclosing their autistic identity did not always ameliorate this.

‘*They’re just kind of like . . . well, if you can’t meet the requirements of the course, then . . . maybe you shouldn’t do it*’. (P9)

### GET9: reflections on diagnostic labelling

Most participants considered their BPD diagnostic label to be a misdiagnosis, rather than concurrent. They reported a sense of relief when this feeling was validated by autism specialist clinicians.

‘*The psychiatrist . . . said it clearly wasn’t appropriate to have me diagnosed with BPD*’. (P7)

Despite this recognition, seeking formal de-diagnosis was challenging.

‘*It was a really hard label to get rid of once it’s there*’. (P10)

This process forced participants to re-face feelings of powerlessness and worries about being labelled as difficult.

‘*You can’t challenge it, because if you challenge it in any way, you’re just being difficult*’. (P3)

### GET10: helping others

Participants’ experiences had instilled in them a desire to promote social justice. They used their lived experience to help advocate for an empower others – where possible, preventing similar trauma from re-occurring.

‘*You’ve just got to basically fight the mental health system*’. (P2)

‘*You do have a right to have a say in your care*’. (P7)

Participants stressed the importance of persistence, if one is to successfully avoid misdiagnosis and any associated harms.

‘*Don’t drop the topic*’. (P7)

‘*Try and find a private psychiatrist who specialises in autism*’. (P3)

All participants were now involved in a sector that deals with supporting vulnerable people. Nine were involved in delivering healthcare – mostly mental healthcare.

## Discussion

Our study has explored the experiences of autistic adults who were previously diagnosed with BPD/EUPD. In most cases, BPD/EUPD was reported to be a misdiagnosis. This misdiagnosis carried stigma and led to deeply harmful experiences for our participants. While they did not identify with the diagnosis, they felt powerless to challenge it. In contrast, receiving an autism diagnosis was ‘life changing’. While this did not solve everything, it was deeply validating. It allowed them to be their truly authentic selves – shifting the focus away from ‘treatment’ and changing who they are to suit others, towards acceptance of their differences. This significantly improved their mental health. Reflecting back on their experiences, participants wanted to help prevent others from experiencing similar trauma.

Of particular note, were our participants’ feelings that their BPD/EUPD diagnosis was overtly harmful. Not only did this overshadow their autistic status, but it introduced a great deal of stigma. We can unpack the negative impact of a BPD/EUPD diagnosis on their sense of self using Cooley’s theory of the ‘looking glass self’. This theory posits that we subconsciously consider how we appear to others, how they judge us based on this and then we incorporate this into our self-concept (Shaffer, 2005). Our participants recalled the sense of blame associated with a BPD/EUPD diagnosis, leading them to question who they were as people. This, combined with increasing feelings of helplessness, may have contributed to the worsening mental health they reported.

The BPD/EUPD diagnosis also led to our participants receiving treatment approaches that encouraged masking behaviours, alongside inpatient admissions that did not adjust for sensory differences. Masking is a vital consideration, as this has been identified as a risk factor for suicide in autistic adults (Cassidy et al., 2018). This would support our participants’ views around the negative impacts on their wellbeing, alongside the positive impacts of being able to unmask following their autism diagnosis. This reinforces the importance of identifying autism early, avoiding misdiagnosis. Our participants, however, recalled a variety of differences that could indeed overlap between autism and BPD/EUPD. For instance, suicidality and self-harm (American Psychiatric Association, DSM-5 Task Force, 2013; Fitzgerald, 2005; Trubanova et al., 2014). In addition, abuse or trauma is considered one of the risk factors for BPD/EUPD (American Psychiatric Association, DSM-5 Task Force, 2013). On the contrary, autistic people are prone to becoming victims of abuse and trauma (American Psychiatric Association, DSM-5 Task Force, 2013; Brown et al., 2017; Brown-Lavoie et al., 2014; Ohlsson Gotby et al., 2018). Furthermore, autistic shutdowns often occur due to accumulated autistic burnout, sensory overload or exhaustive masking (Mantzalas et al., 2022; Phung et al., 2021). However, in the case of our participants, these were mistaken for dissociative episodes, providing confirmatory bias to support BPD/EUPD diagnoses. This has also been found in the wider literature (Lyssenko et al., 2018; Reuben & Parish, 2022; Schalinski et al., 2015; Scott & Baron-Cohen, 1996).

While there is no consensus among clinicians around the co-occurrence of BPD/EUPD and autism, national guidelines recognise the potential for co-occurrence (NICE, 2021). Our participants were, however, mostly misdiagnosed with BPD/EUPD, which was later de-diagnosed on diagnosing autism. However, de-diagnosis was a difficult journey for many. This is an important journey to understand, as an increasing number of people with a BPD/EUPD diagnosis are receiving an autism diagnosis later in life (Russell et al., 2022). Our participants views around the ease of (and bias towards) giving BPD/EUPD diagnosis, compared to a reluctance to diagnose autism, may offer some possible explanation for this trend. This is supported by the wider literature, which shows that psychiatrists receive inadequate training in this area and do not feel confident in recognising or diagnosing autism (Crane et al., 2019). Until relatively recently, even the existence of autistic people working in psychiatry was largely unrecognised (McCowan et al., 2022). Autism awareness within psychiatry has, however, begun to increase exponentially in recent years, as has the acceptance and championing of lived experience (Department of Health and Social Care, 2021).

The disproportionately cis-female representation in our study is also important. This may imply gender bias in the initial diagnostic labelling of BPD/EUPD. This is indeed supported by literature (Bjorklund, 2006; Skodol, 2003). Furthermore, autistic women are more likely to mask their differences (Milner et al., 2019). This combination could be dangerous. Those most likely to be misdiagnosed with BPD/EUPD, and thus exposed to treatments promoting masking, may be those most likely to mask at baseline – potentially increasing the risk of harm to their self-concept and experiences of suicidality (Bradley et al., 2021; Cassidy et al., 2020). This is certainly in keeping with the experiences of our participants.

Autism awareness has been increasing among the general public in recent years (Department of Health and Social Care, 2021; Dillenburger et al., 2013). Our participants were often encouraged by clinicians or colleagues to consider an autism assessment. However, our participants felt it was challenging to convince their psychiatrists to make the referral. They discussed a sense of power dynamics between patients and doctors. They felt that the traditional patriarchal doctor–patient relationship left them feeling powerless and compelled to accept diagnoses with which they did not agree. Indeed, many patients may be unaware of their rights. For instance, patients can ask for a second opinion. This lack of awareness may contribute to their sense of powerlessness. However, one participant did express that patients have the right to decide on their care. This may reflect a changing culture in patient–doctor relationships within the NHS.

Scepticism among mental health professionals about autism in the BPD/EUPD group has been shown to be a significant issue in the wider literature (de Broize et al., 2022; Murphy et al., 2022). Even if the clinicians make the referral, people are then subject to waiting lists of up to several years (British Medical Association, 2019). According to Hickey et al., an autism diagnosis validates challenges, gives insight into the sense of self and empowers people (Hickey et al., 2018). Our participants were able to relate to this and felt relieved on receiving an autism diagnosis. Most of our participants also reported significant improvement in their mental health. This is likely due to the sense of validation and gained insight. In addition, an autism diagnosis was helpful in social life, especially in educational settings and the workplace. It has been suggested elsewhere that detection of autism itself may also act as a form of ‘primary prevention of mental ill health’ in autistic people (Shaw, Doherty, et al., 2022). It is worth noting here, however, that while not reported by our own participants, some people with a BPD diagnosis may also find this validating – and it is not our intention to invalidate such an experience.

An autism label does, however, carry an associated stigma (Aube et al., 2021; Salleh et al., 2020; Turnock et al., 2022). However, our participants explained how this was quite different from the stigma attached to BPD/EUPD. In our participants, stigma around autism came in the form of scepticism about the diagnosis, linked to deficit-based stereotypes, which led to questions around their autonomy. This was in direct contrast to the perceived stigma around BPD/EUPD, which focused on their status as broken human beings or the risks they posed to others – allowing the system to neglect or leave them behind. These experiences can be explained using the theoretical concept of ‘ethical loneliness’ (Rosenblatt, 2018). This can be defined as ‘*the experience of being abandoned by humanity compounded by the experience of not being heard*’ (Rosenblatt, 2018). In the case of our participants, they reflected on how their experiences of dismissal or neglect following a BPD/EUPD diagnosis were contextualised within wider system issues (e.g. funding). They felt that this new diagnostic label allowed others to dismiss their voices and needs. Viewing this through the lens of ethical loneliness, one can see how their exclusion was felt to be for the betterment of others, through the preservation of scarce resources. Indeed, wider studies do report that BPD/EUPD is often considered the most stigmatised mental health diagnosis (BPD Community 2016). It is frequently associated with increased stigma from mental health staff (Bodner et al., 2015; McKenzie & Hogg, 2022), supporting the experiences of our participants. Future study is needed to explore the experiences of mental health professionals working in this area, to shed light on their perspectives here.

Our participants eventual dedication of their lives to improving the system for others is also important. This refutes the traditional misassertion that autistic people do not feel empathy. In fact, more modern literature suggests that autistic people may feel more empathy than non-autistic counterparts (Fletcher-Watson & Bird, 2020) – just displayed differently (Shaw et al., 2023). This is supported by the theory of the ‘double empathy problem’ and studies of cross-neurotype communication differences (Crompton et al., 2020; Milton, 2012). Through using their own lived experience to promote positive change, our participants were fostering epistemic justice within the system.

### Strengths and limitations

The phenomenological nature of our study design helps us to understand the experiential account of complex phenomena. It provides an opportunity to do a detailed and nuanced analysis of each participant’s perspective. In addition, it allows cross-case analysis for convergence and divergence between participants. The study design does not seek to be generalisable. The fact the study included 10 participants, increases the richness of the data, promoting meaningful interpretation of their accounts. This is also an epistemic strength, as it allows their lived experience to impact academic spaces. It is worth noting that participants may have been more likely to take part in this study if they had strong feelings they wished to portray. This does not, however, invalidate their feelings and experiences. The purpose of interpretive phenomenology is not to generalise, but to dig into the meaning behind the experiences of those who did participate – shedding light on their reality, as they experienced it.

Our study also benefits from our own positionality as researchers. For example, the lead researcher is a practising psychiatrist worked in a specialist personality disorder service and the Principal Investigator is autistic. This positioning was key within our methodological approach, allowing us to draw on our own insights to aid our interpretive analysis. However, we acknowledge that our positioning could also be considered a limitation, depending on our readers’ own philosophical beliefs.

Our choice of data collection methods may be a limitation. For example, this required participants to speak English and to be comfortable with the social nature of an interview. Our findings may, therefore, overlook the important experiences of autistic people who are non-speaking, or who might have found an interview inaccessible. Future cross-sectional study in this area might help to overcome this in some ways, while also exploring the potential for generalisability of our findings here.

## Conclusion

Our study has explored the experiences of autistic adults who were previously diagnosed with BPD/EUPD. The misdiagnosis with BPD/EUPD carried great stigma and led to harmful experiences. In contrast, receiving an autism diagnosis was ‘life changing’. This was deeply validating and allowed them to be their true authentic selves. This shifted the focus away from ‘treatment’ and changing who they are to suit others, towards acceptance of their differences and neurotype. This significantly improved their mental health. Reflecting back on their experiences, participants wanted to help prevent others from experiencing similar trauma. Subsequently, almost all were now involved with healthcare services in a supportive manner.

Misdiagnosis is preventable. We call for improved autism training for psychiatrists alongside consideration for automatic autism screening when adults are diagnosed with BPD/EUPD.

Future research is now needed to consider the generalisability of our findings and also to explore the perspectives of clinicians working in this area.

## Supplemental Material

sj-docx-1-aut-10.1177_13623613241276073 – Supplemental material for The experiences of autistic adults who were previously diagnosed with borderline or emotionally unstable personality disorder: A phenomenological study Supplemental material, sj-docx-1-aut-10.1177_13623613241276073 for The experiences of autistic adults who were previously diagnosed with borderline or emotionally unstable personality disorder: A phenomenological study by Bruce Tamilson, Jessica A Eccles and Sebastian C K Shaw in Autism
