# Supplementary material for: The experiences of autistic adults who were previously diagnosed with borderline or emotionally unstable personality disorder: A phenomenological study
Source: Autism. 2024 Sep 11;29(2):504–17. doi: 10.1177/13623613241276073 (PMC11816473; doi:10.1177/13623613241276073)
Supplement: sj-docx-1-aut-10.1177_13623613241276073 – Supplemental material for The experiences of autistic adults who were previously diagnosed with borderline or emotionally unstable personality disorder: A phenomenological study [file sj-docx-1-aut-10.1177_13623613241276073.docx]

**Interview topic guide**

| **Main Questions** | **Possible additional Questions** |
| --- | --- |
| Tell me a little about yourself |  |
| Tell me about your personality disorder diagnosis | Things to consider and possibly explore further:   - What led to you receiving this diagnosis? - How were you diagnosed and by whom? - Did you agree with it? - Did you tell people about it? If so, did they treat you differently? - How did the label impact on you emotionally? |
| Tell me about your later journey to an autism diagnosis. (Or self-realisation if not formally diagnosed) | Things to consider and possibly explore further:   - What led to you considering that you might actually be autistic? - Why did you feel this was a better fit than the personality disorder label? - How did you approach getting a diagnosis? Who was this from? How difficult was it to get? - Did you tell people about it? If so, did they treat you differently? - How did the label impact on you emotionally? |
| What do you think the main differences have been for you, knowing you are autistic, compared to your previous personality disorder label? | Things to consider and possibly explore further:   - Has this been a positive or negative experience? And why? - Do you feel there is any difference in stigma, or the way people treat or perceive you? |
| Have you noticed any differences in your mental health since receiving your autism diagnosis? (Or self-realisation) | Things to consider and possibly explore further:   - Have you noticed yourself needing to seek support for your mental health less or more often? Why do you think that might be? |
| Why do you think your autism diagnosis was initially missed? |  |
| Looking back, is there anything that you might have done differently? |  |
| Looking back, is there anything the clinical services could have done differently? |  |
| What advice might you give to anyone living with a borderline or emotionally unstable personality disorder diagnosis who might suspect they are actually autistic? |  |
| Is there anything else you would like to discuss? |  |
